# Geometric distortion in computed tomography scans for radiotherapy: Quantification and quality assurance implications

**DOI:** 10.1002/acm2.70686

**Published:** 2026-07-07

**Authors:** Yunfei Hu, Emma Cai, John Shakeshaft, Marius Arnesen, Tina Gorjiara, Mikel Byrne, Tim Markwell, Ben Archidbald‐Heeren

**Affiliations:** ^1^ Icon Cancer Centre Gosford Gosford New South Wales Australia; ^2^ University of Wollongong Wollongong New South Wales Australia; ^3^ Icon Cancer Centre Concord Concord Repatriation General Hospital Concord New South Wales Australia; ^4^ Icon Cancer Centre Gold Coast Southport Queensland Australia; ^5^ Icon Cancer Centre Toowoomba Rockville Queensland Australia; ^6^ Icon Cancer Centre Norwest Bella Vista New South Wales Australia; ^7^ Icon Cancer Centre South Brisbane Queensland Australia; ^8^ Icon Cancer Centre Hobart Hobart Tasmania Australia

**Keywords:** CT, geometric distortion, MRI, stereotactic radiotherapy

## Abstract

**Background:**

Computed tomography (CT) is widely assumed to be geometrically accurate and is commonly used as the reference standard for quantifying geometric distortion in other imaging modalities, particularly magnetic resonance imaging (MRI). However, geometric distortion in CT has not been comprehensively investigated.

**Purpose:**

The aim of this study was to characterize the performance of a distortion phantom, establish a reference scan and, on this basis, determine and quantify the presence of geometric distortion in CT scans used for radiotherapy (RT) planning.

**Methods:**

A CIRS Model 603A Distortion Phantom was employed to quantify geometric distortion. System reproducibility, as well as the effects of phantom setup, scan parameters, fabrication uncertainty, and temporal variation were systematically evaluated. Additionally, the geometric distortion of CT was compared to that of linac‐based cone‐beam CT (CBCT). Subsequently, geometric distortion magnitudes from eight CT scanners were retrospectively analyzed against both CBCT and a Computer Aided Design (CAD) model.

**Results:**

CBCT demonstrated consistently smaller distortion variation compared with CT. Retrospective analysis revealed that all eight analyzed CT scanners exhibited measurable distortion magnitudes, with mean magnitudes ranging from 0.13 mm to 0.52 mm and maximum distortion magnitudes ranging from 0.45 mm to 1.09 mm when compared to CBCT, or 0.25 mm to 0.82 mm and 0.58 mm to 1.88 mm when compared to the CAD model. Longitudinal couch inaccuracy or non‐linear couch sag may constitute the primary contributors to geometric distortion on CT scanners.

**Conclusions:**

Contrary to common assumptions, CT scans used for radiotherapy planning exhibit measurable geometric distortion across clinically relevant fields of view (FoVs). However, current CT QA methodologies and tolerances may lack sufficient sensitivity to detect submillimeter distortion levels relevant to stereotactic radiotherapy (SRT). Recognition and rigorous quantification of CT geometric distortion are essential to inform risk assessment and margin design in high‐precision RT.

## 1 INTRODUCTION

The purpose of radiotherapy (RT) is to deliver precise dose to the target while sparing the surrounding healthy tissue. As a result, the geometric accuracy of images used for RT planning is essential, particularly in the context of stereotactic radiotherapy (SRT) where sub‐millimeter margins are employed.^1^ One factor that may compromise the geometric accuracy of RT treatment is geometric distortion of the RT planning image.

Both computed tomography (CT) and magnetic resonance imaging (MRI) are common imaging modalities used in RT planning. CT provides information that assists in not only the delineation of internal and external anatomical contours, but also the creation of a density map required for precise dose calculations.^2^, ^3^ MRI, on the other hand, provides superior soft tissue contrast and non‐ionizing characteristics.^4^, ^5^

Although the geometric distortion of MRI has been well quantified by multiple studies,^6^, ^7^, ^8^, ^9^, ^10^ similar research on CT images remains scarce. Instead, CT scans have been commonly employed as the reference scan to quantify geometric distortion in MRI and other imaging modalities,^11^, ^12^, ^13^, ^14^, ^15^ based on the inherent assumption that there is no geometric distortion in CT images. Nonetheless, there are several factors that can contribute to a non‐uniform distribution of geometric distortion in CT images, including but not limited to gantry tilt miscalibration, reconstruction‐related issues, and non‐linear bending of the CT couch in the longitudinal direction.^16^ Well‐established quality assurance (QA) guidelines for CT simulators and simulation processes, such as those proposed by the American Association of Physicists in Medicine (AAPM),^17^ have listed in‐plane spatial integrity as a compulsory daily check. However, it is usually performed by simply measuring the outer dimensions of a phantom with known geometry.^18^ This QA approach provides restricted accuracy as well as limited information regarding the distribution of geometric distortion within the field of view (FoV).

**FIGURE 1:**
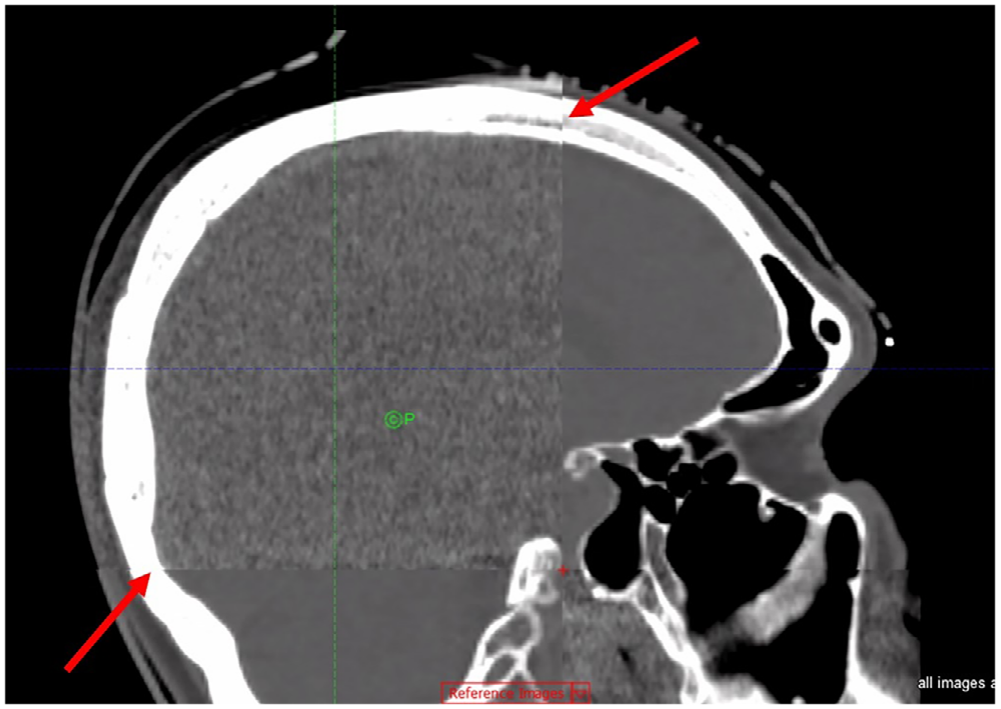
Sagittal view of the CT and CBCT scans of a cranial patient. The two scans have been registered using rigid registration and displayed using the same window level (bone). The interval between the acquisitions of the two scans is less than a week. Nonetheless, mismatch in the skull is observed, highlighted by the red arrows. Given the rigidity of the skull, this mismatch likely originates from image distortion rather than anatomical variation.

The authors’ organization is composed of multiple regional centers, many of which do not have a dedicated CT scanner but rely on collaborations with local radiology departments to perform CT simulation for RT planning. Under these circumstances, the risk of geometric distortion is increased due to the mixed use of the CT scanner for RT scans, which utilize a 0 degree gantry tilt, a hard flat couchtop, as well as dedicated protocols, compared with diagnostic scans, which employ various gantry tilts, a soft dished couchtop, and protocols that are typically adjusted on the fly.^19^ Additionally, in clinical practice, geometric misalignment between CT and cone‐beam computed tomography (CBCT) has been observed in anatomical regions where anatomical changes are unlikely to occur, such as the skull as shown in Figure 1, highlighting potential geometric distortion. Therefore, it is critical to determine the presence of geometric distortion in CT scans, particularly those used for SRT planning.

In this study, the CIRS Model 603A Distortion Phantom for Stereotactic Radiosurgery (SRS) (CIRS Inc., Norfolk, VA, USA; part of Mirion Technologies, Inc.), subsequently referred to as the CIRS distortion phantom, was employed to determine and quantify geometric distortion on CT scanners. The aim of the study was to provide references for the quantification of a geometric distortion map within the FoV of CT scans, thereby informing margin design and uncertainty analysis in precise RT.

## 2 METHODS

### 2.1 CIRS distortion phantom

Featuring a tissue‐equivalent, anthropomorphic design, the CIRS distortion phantom can be imaged using X‐ray, CT, and MRI. The entire inter‐cranial portion of the skull volume is filled with an orthogonal 3D grid of 2.5 mm diameter rods spaced 10 mm (I–S), 10.5 mm (A–P), and 11 mm (L–R) apart. Five extended axis‐rods intersect at the reference origin of the grid, the end of which is fitted with CT/MR markers for co‐registration.^20^

Once a scan of the CIRS distortion phantom is acquired, the dataset is imported into the DistortionCHECK™ software (Sun Nuclear, Middleton, WI, USA; part of Mirion Technologies, Inc.) for analysis.^21^ Either the default template used for phantom fabrication, referred to as the Computer Aided Design (CAD) model (i.e., a .csv file containing a predefined set of reference coordinates), or a user‐selected scan can be designated as the reference dataset. Upon import of a test scan, the grid intersection points are automatically identified using an intensity‐based centroid detection method as described by Stanescu et al..^22^ A rigid registration is then performed between the reference and acquired datasets, followed by resampling with interpolation to establish spatial correspondence and generate a three‐dimensional distortion field.^15^ Following registration, the software determines the X, Y, and Z coordinates of each marker centroid in both the reference and the acquired scans. The geometric distortion magnitude (in mm), denoted as ℰ, is subsequently calculated as the Euclidean distance between corresponding marker coordinates using the following equation:

$$E=\sqrt{{\left(X_{i}-X_{ref}\right)}^{2}+{\left(Y_{i}-Y_{ref}\right)}^{2}+{\left(Z_{i}-Z_{ref}\right)}^{2}}$$

**FIGURE 2:**
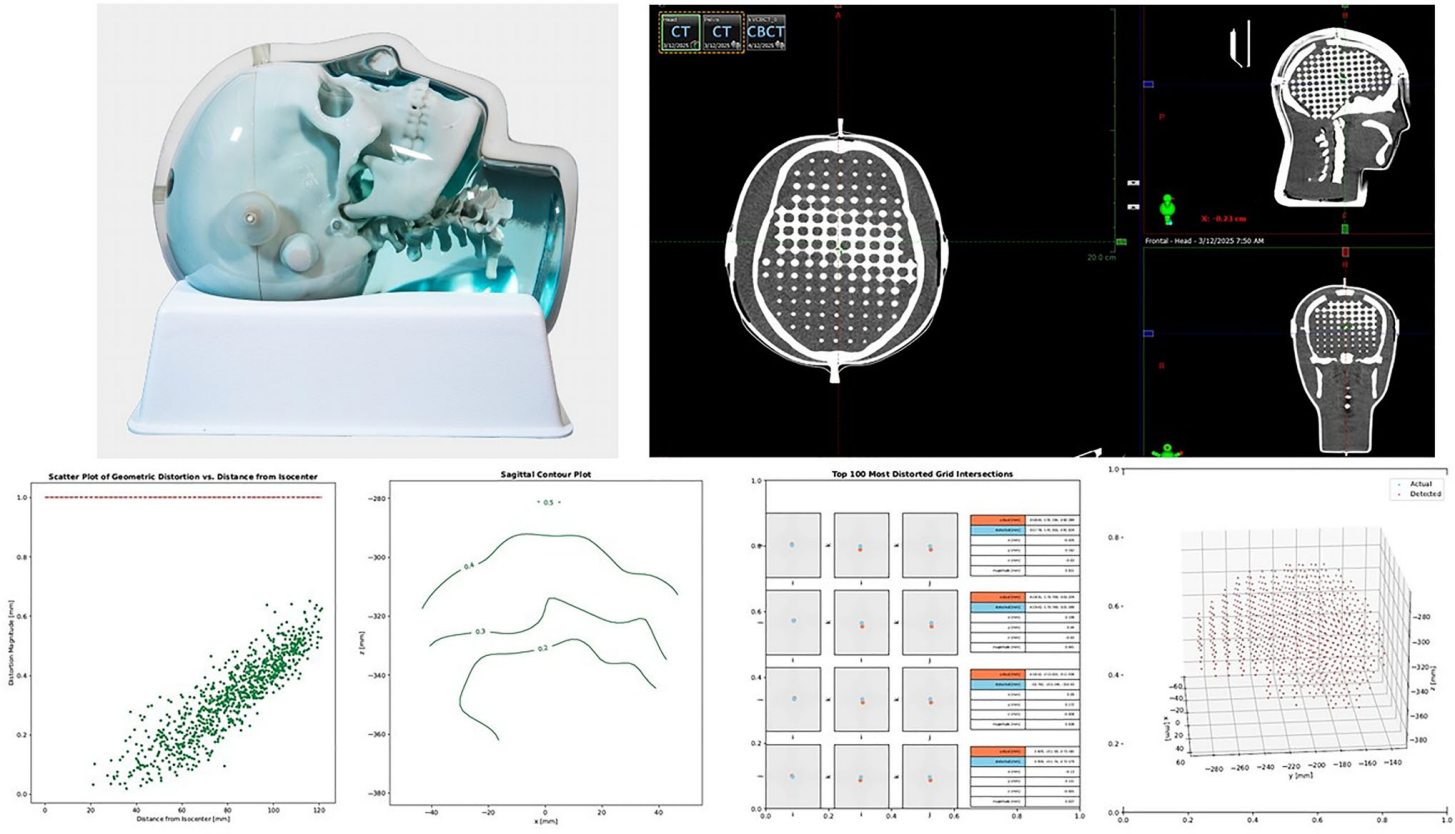
Top left: A photo of the CIRS distortion phantom (https://www.sunnuclear.com/uploads/documents/datasheets/603GSPhantom_080123.pdf; accessed on the 22^nd^ of April, 2026); Top right: CT scan of the CIRS distortion phantom; Bottom, from left to right: Scatter plot of geometric distortion vs. distance from isocenter, sagittal contour plot, top 100 most distorted grid intersections, and 3D vector map of geometric distortion, provided in the report generated by the DistortionCHECK™ software.

By identifying the centroid of the markers, the distortion magnitude can be calculated with a spatial precision that is not limited by the voxel resolution. A detailed report is then generated by the DistortionCHECK™ software,^15^, ^23^ as shown in Figure 2.

### 2.2 CT and CBCT equipment

A total of eight CT scanners and four linacs with CBCT capability were included in this study. No specific eligibility criteria were applied to the selection of imaging devices. Instead, the scanners and linacs were selected using a convenience sampling approach. This was considered appropriate, as imaging protocols for both CT and CBCT are standardized across departments within the organization, thereby ensuring consistency in acquisition parameters and facilitating meaningful comparison across devices. Routine QA was completed and passed on all CT and CBCT devices following standard guidelines.^17^, ^24^ All scanners and linacs were located within RT departments where ambient environmental conditions (e.g., temperature, pressure, and humidity) are tightly controlled and maintained within narrow tolerances throughout the year. Detailed information of the CT and CBCT devices is listed in Table 1. All CT and CBCT images were reconstructed with a matrix size of 512 × 512. For both CT and CBCT acquisitions, the scan length was adjusted to fully encompass the entire CIRS distortion phantom, which utilized a z‐axis collimation length of 18.6 cm at the isocenter for CBCT imaging.

**TABLE 1:** Details of CT and CBCT devices.

Equipment ID	Modality	Vendor	Model	kVp	Use
CT_1	CT	Siemens	Go.Sim	120	RT Only
CT_2	CT	GE	Lightspeed	120	Radiology & RT
CT_3	CT	Siemens	Go.Sim	120	RT Only
CT_4	CT	Canon	Aquilion	120	Radiology & RT
CT_5	CT	Canon	Aquilion	120	Radiology & RT
CT_6	CT	Canon	Aquilion	120	Radiology & RT
CT_7	CT	Siemens	Go.Sim	120	RT Only
CT_8	CT	Siemens	Go.OpenPro	120	RT Only
CBCT_1	CBCT	Varian	Truebeam	100	RT Only
CBCT_2	CBCT	Varian	Truebeam	100	RT Only
CBCT_3	CBCT	Varian	Truebeam	100	RT Only
CBCT_4	CBCT	Elekta	Versa	100	RT Only

### 2.3 Preliminary reproducibility assessment

To verify the consistency of the proposed DistortionCHECK™ workflow in detecting submillimeter uncertainties, a preliminary reproducibility assessment was performed. Specifically, the same phantom was scanned two consecutive times on CT_3 using the RT Head protocol (which entails a FoV of 400 mm and a slice thickness of 1.0 mm) and an identical setup. Subsequently, using the first scan as the reference dataset, the geometric distortion magnitudes of the second scan were calculated. The geometric distortion magnitudes obtained from these analyses were then ranked in ascending order. The 95th percentile value was subsequently determined and reported as the measurement uncertainty associated with the DistortionCHECK™ workflow. Additionally, the datasets were reanalyzed three times within the DistortionCHECK™ software to assess inter‐analysis repeatability.

### 2.4 Effects of phantom setup and scan parameters on distortion analysis

To understand the effects of phantom setup (i.e., rotation/roll/pitch and translational shifts) and scan parameters (i.e., FoV size and slice thickness) on the accuracy of distortion analysis, Scans 1 – 5 listed in Table 2 were acquired. All scans were acquired on the same CIRS distortion phantom on CT_3. All datasets in this section were compared against the default CAD model.

**TABLE 2:** Details of test scans.

2.4 Effects of Phantom Setup and Scan Parameters			
Scan ID	FoV (mm)	Slice Thickness (mm)	Rotation/Roll/Pitch (°) & Translation (mm)
1	400	1.0	0°, 0 mm
2	400	1.0	2°, 0 mm
3	400	1.0	5°, 0 mm
4	400	1.0	0°, 1 mm in the cranio‐caudal direction
5	750	2.0	0°, 0 mm

2.5(a) Effects of Fabrication Uncertainty			
Scan ID	FoV (mm)	Slice Thickness (mm)	Internal Phantom ID
6	400	1.0	Phantom 1
7	400	1.0	Phantom 2
8	400	1.0	Phantom 3

2.5(b) Effects of Temporal Variation			
Scan ID	FoV (mm)	Slice Thickness (mm)	Date of Scan (dd/mm/yy)
9	400	1.0	16/12/24
10	400	1.0	09/12/25

2.6 Comparison of the Geometric Distortion of CT and CBCT			
Scan ID	FoV (mm)	Slice Thickness (mm)	Equipment ID
11	400	1.0	CT_1
12	400	1.0	CT_2
13	400	1.0	CT_3
14	400	1.0	CT_4
15	262	1.0	CBCT_1
16	262	1.0	CBCT_2
17	262	1.0	CBCT_3
18	262	1.0	CBCT_4

### 2.5 Effects of fabrication uncertainty and temporal variation on distortion analysis

To assess potential variations in marker locations among different phantoms, Scans 6 – 8 listed in Table 2 were acquired using the RT Head CT protocol across multiple CIRS distortion phantoms. All phantoms were scanned using an identical set up in a vacbag (Klarity, Heath, OH) to minimize the rotation/roll/pitch of the phantom. All scans were acquired consecutively on CT_8.

Additionally, it is necessary to determine whether the detected geometric distortion changes with time. This can be due to changes in either the performance of the CT scanner or marker locations within the phantom. For this purpose, Scans 9 and 10 listed in Table 2 were acquired on the same phantom using the RT Head protocol on CT_3, but roughly one year apart.

All datasets in this section were compared against the default CAD model.

### 2.6 Comparison of the geometric distortion of CT and CBCT

In this section, the geometric distortion magnitudes of two imaging modalities, that is, CT and CBCT, were compared. Specifically, the same CIRS distortion phantom was scanned on multiple CTs and linac‐based CBCTs. A vacbag (Klarity, Heath, OH) was employed during setup to minimize the rotation/roll/pitch of the phantom. Details of the acquired scans, that is, Scans 11 – 18, are listed in Table 2. For both CT and CBCT scans, the default RT Head protocol was used, which entails a FoV of 400 mm for CT scans and 262 mm for CBCT scans. Under a matrix of 512 × 512, this equates to a voxel size of approximately 0.78 × 0.78 × 1.00 mm^3^ for CT scans and 0.51 × 0.51 × 1.00 mm^3^ for CBCT scans.

All datasets in this section were compared against the default CAD model.

### 2.7 Quantification of geometric distortion in CT

In this section, the geometric distortion magnitudes of eight CT scanners, that is, CT_1 to CT_8, were retrospectively analyzed. All scans were acquired with the default RT Head protocol, which features a FoV of 400 mm and a slice thickness of 1.0 mm. Scans in this section were compared to both CBCT and the CAD model.

## 3 RESULTS

### 3.1 Preliminary reproducibility assessment

Compared with the first scan, the mean and maximum distortion magnitudes of the second scan acquired from the same phantom using an identical protocol and setup were 0.06 mm and 0.22 mm, respectively. When the values were arranged in ascending order, the 95th percentile geometric distortion magnitude (816th value out of 859 points) was 0.13 mm. This value was subsequently adopted as the measurement uncertainty of the DistortionCHECK™ workflow in the following sections.

Repeated analysis of the same dataset yielded identical results, confirming the inter‐analysis reproducibility of the DistortionCHECK™ software.

### 3.2 Effects of phantom setup and scan parameters on distortion analysis

**FIGURE 3:**
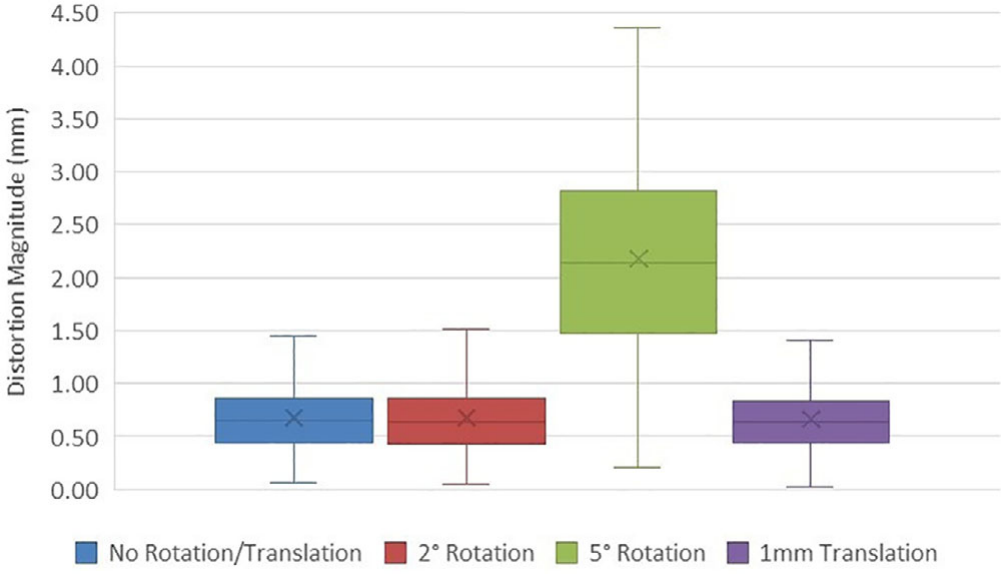
Analysis of the effects of setup uncertainty (rotation and translational shifts) on the accuracy of geometric distortion analysis. All other confounding factors were controlled. The CAD template was used as the reference.

The effects of phantom setup on measured distortion magnitudes are depicted in Figure 3. Specifically, a 5° rotation of the CIRS distortion phantom resulted in a 1.51 mm increase (2.18 mm vs. 0.67 mm) in the mean distortion magnitude and a 2.65 mm increase (4.36 mm vs. 1.71 mm) in the maximum distortion magnitude compared to the scan acquired with no rotation. In contrast, no obvious differences were observed between scans acquired with either a small degree of rotation or a small translational shift and the scan without rotational or translation shifts. The mean and maximum distortion magnitudes were 0.67 mm and 1.71 mm for the scan without rotation or translation; 0.67 mm and 1.76 mm for the scan with a 2° rotation; and 0.66 mm and 1.71 mm for the scan with a 1 mm translation, respectively.

According to the results evaluating the effects of scan parameters, the scan acquired with a 750 mm FoV and 2 mm slice thickness only demonstrated slightly higher distortion magnitudes than the scan acquired with a 400 mm FoV and 1 mm slice thickness. The mean distortion magnitudes were 0.69 mm and 0.67 mm, respectively, while the maximum distortion magnitudes were 1.94 mm and 1.71 mm, respectively.

### 3.3 Effects of fabrication uncertainty and temporal variation on distortion analysis

**FIGURE 4:**
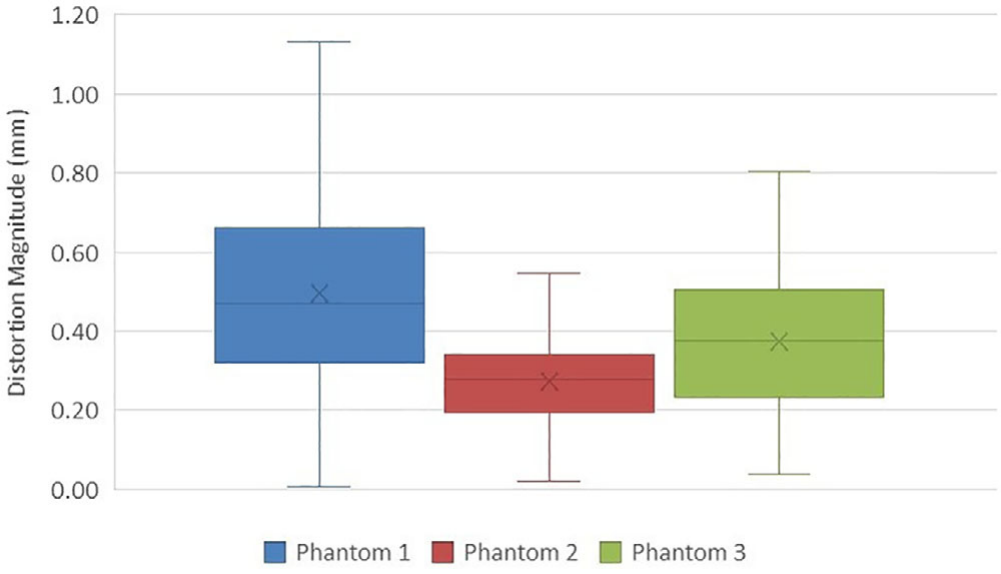
Analysis of fabrication uncertainty by scanning three different phantoms on the same CT. All other confounding factors were controlled. The CAD template was used as the reference.

Results from the test investigating fabrication variations among three different phantoms are shown in Figure 4. Measurable differences in distortion magnitude are observed among Phantom 1 (mean = 0.50 mm, max = 1.13 mm), Phantom 2 (mean = 0.27 mm, max = 0.63 mm), and Phantom 3 (mean = 0.37 mm, max = 0.92 mm).

Alternatively, distortion magnitudes of the same phantom scanned on the same CT but roughly one year apart revealed minimal differences. Specifically, the mean and maximum distortion magnitudes for the scan acquired on 16/12/2024 were 0.68 mm and 1.74 mm, respectively, while for the scan acquired on 09/12/2025, these values were 0.67 mm and 1.78 mm, respectively.

### 3.4 Comparison of the geometric distortion of CT and CBCT

**FIGURE 5:**
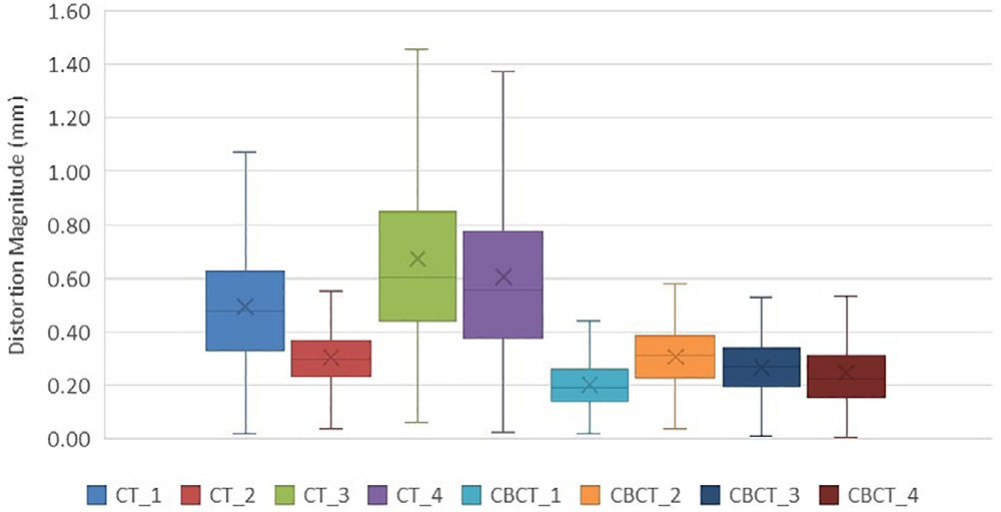
Determination of the imaging modality for reference scans by comparing distortion variation of the same phantom across four CTs and four CBCTs. The CAD template was used as the reference.

The results of testing the variability of distortion magnitudes across different CT and CBCT systems are shown in Figure 5. In most cases, the CBCT systems show consistently smaller geometric distortion magnitudes against the CAD model compared to the CTs.

### 3.5 Quantification of geometric distortion in CT

**FIGURE 6:**
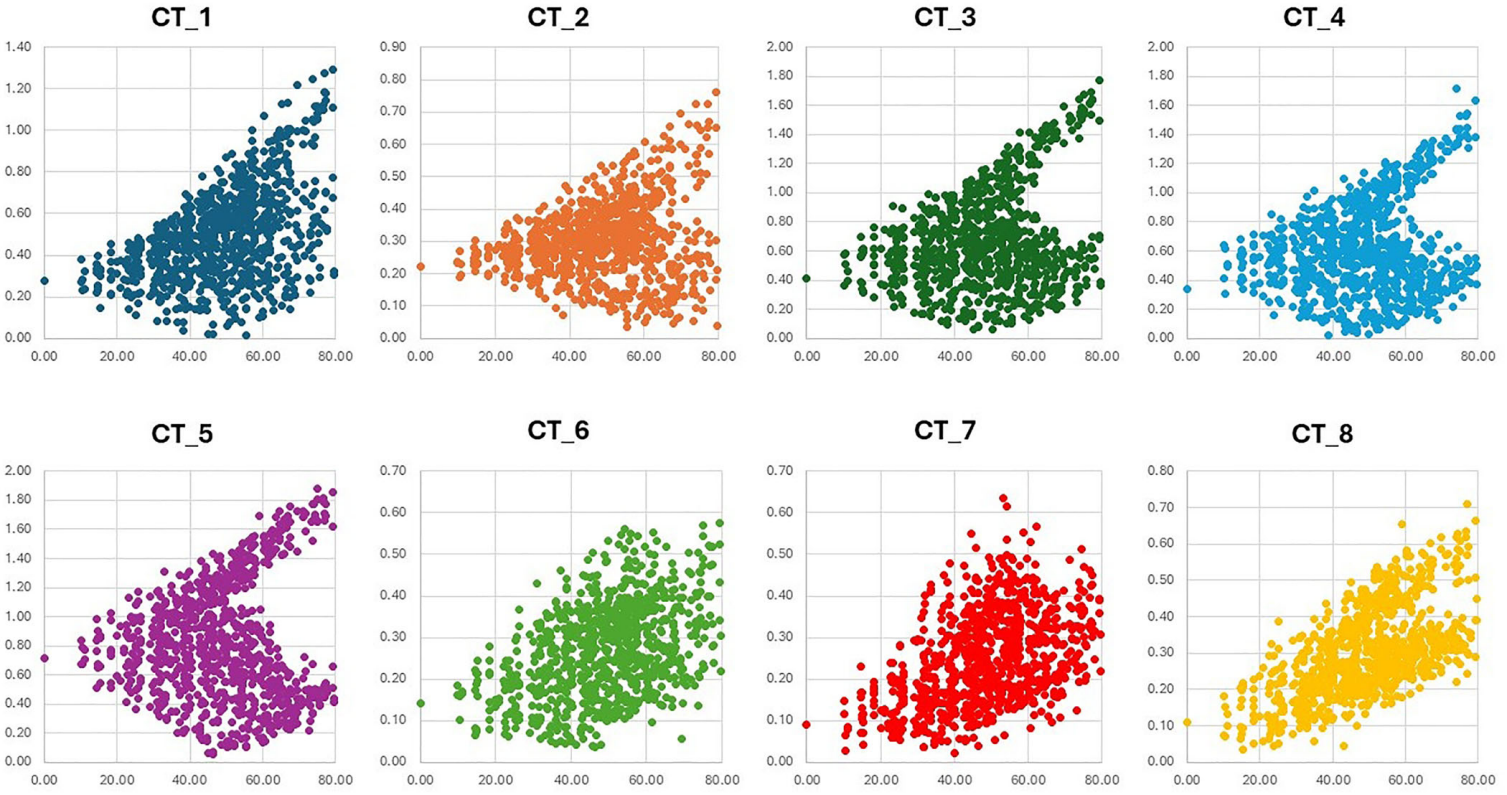
Scatter plots of distortion magnitudes against distance from isocenter for eight CTs, using the CAD model as reference. X‐axis: Distance from isocenter (mm); Y‐axis: Distortion magnitude (mm).

**FIGURE 7:**
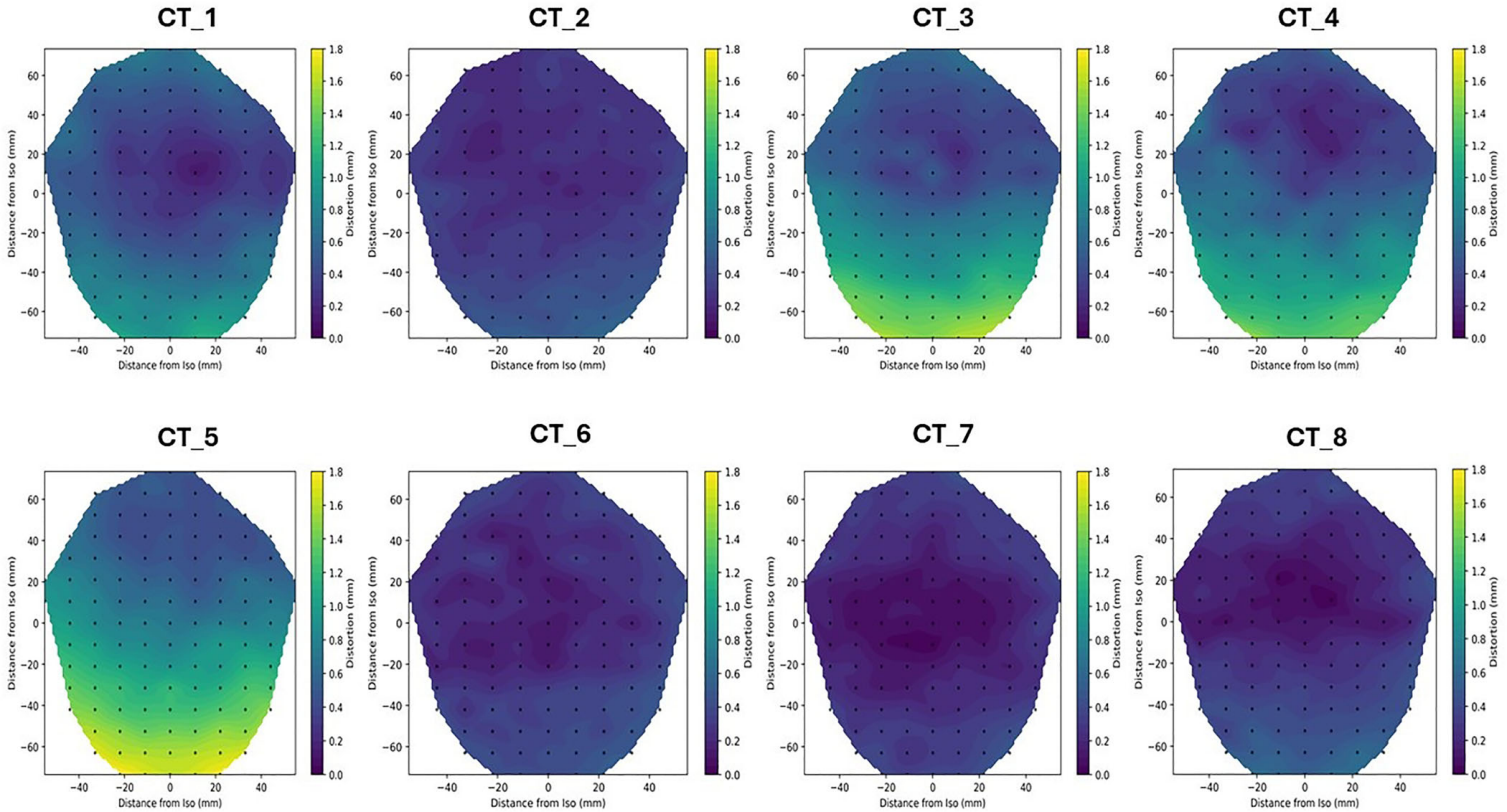
Contour plots of distortion magnitudes at the central axial slice (Z = 0) for eight CTs, using the CAD model as reference. X‐axis: Distance from isocenter (mm) in the L‐R direction; Y‐axis on the left: Distance from isocenter (mm) in the A‐P direction; Y‐axis on the right (color bar): Distortion magnitude (mm). Black dots denote sampled measurement points. Colors in between black dots were interpolated using a cubic function.

**FIGURE 8:**
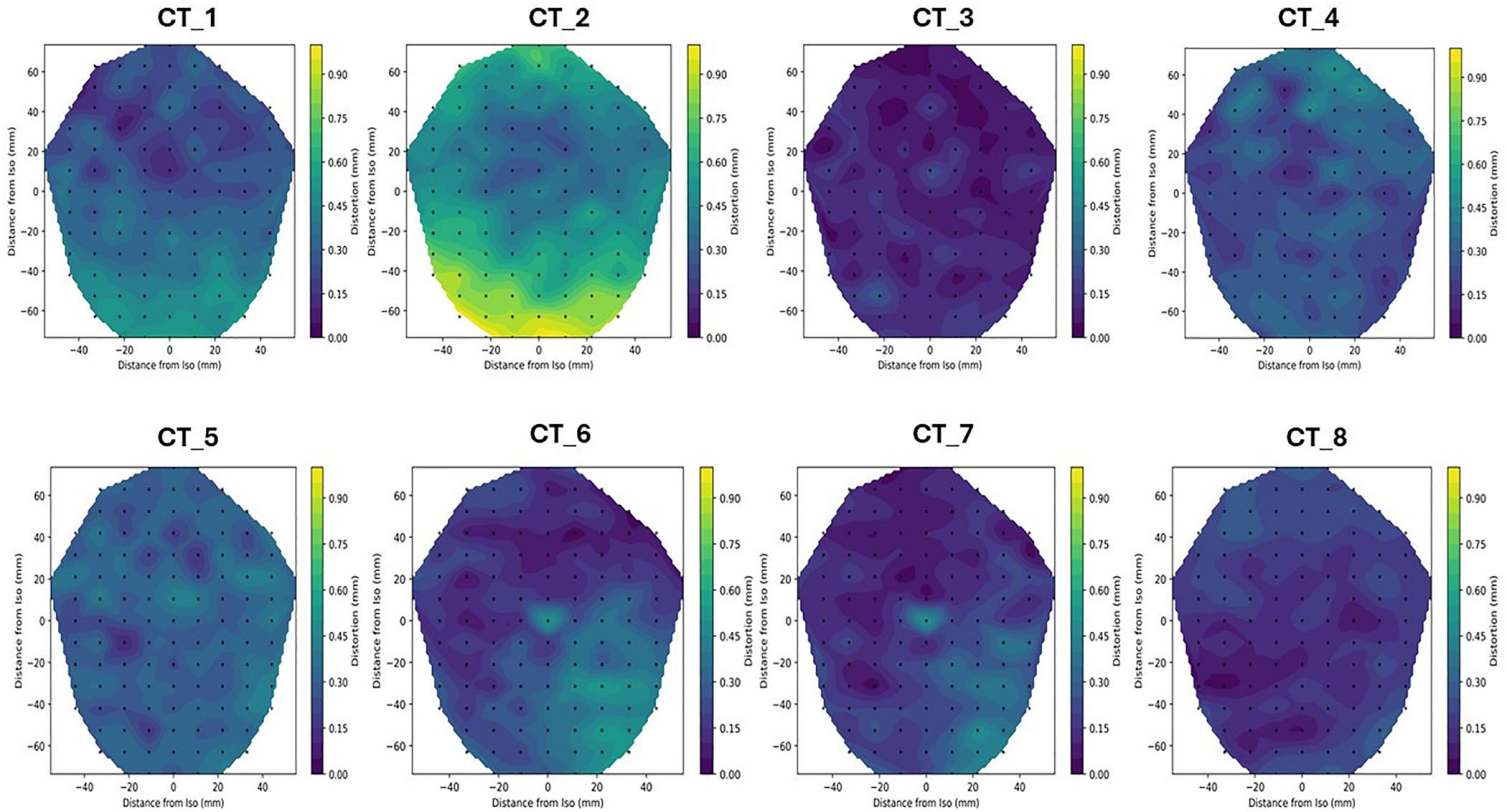
Contour plots of distortion magnitudes at the central axial slice (Z = 0) for eight CTs, using CBCT as reference. X‐axis: Distance from isocenter (mm) in the L‐R direction; Y‐axis on the left: Distance from isocenter (mm) in the A‐P direction; Y‐axis on the right (color bar): Distortion magnitude (mm). Black dots denote sampled measurement points. Colors in between black dots were interpolated using a cubic function.

**FIGURE 9:**
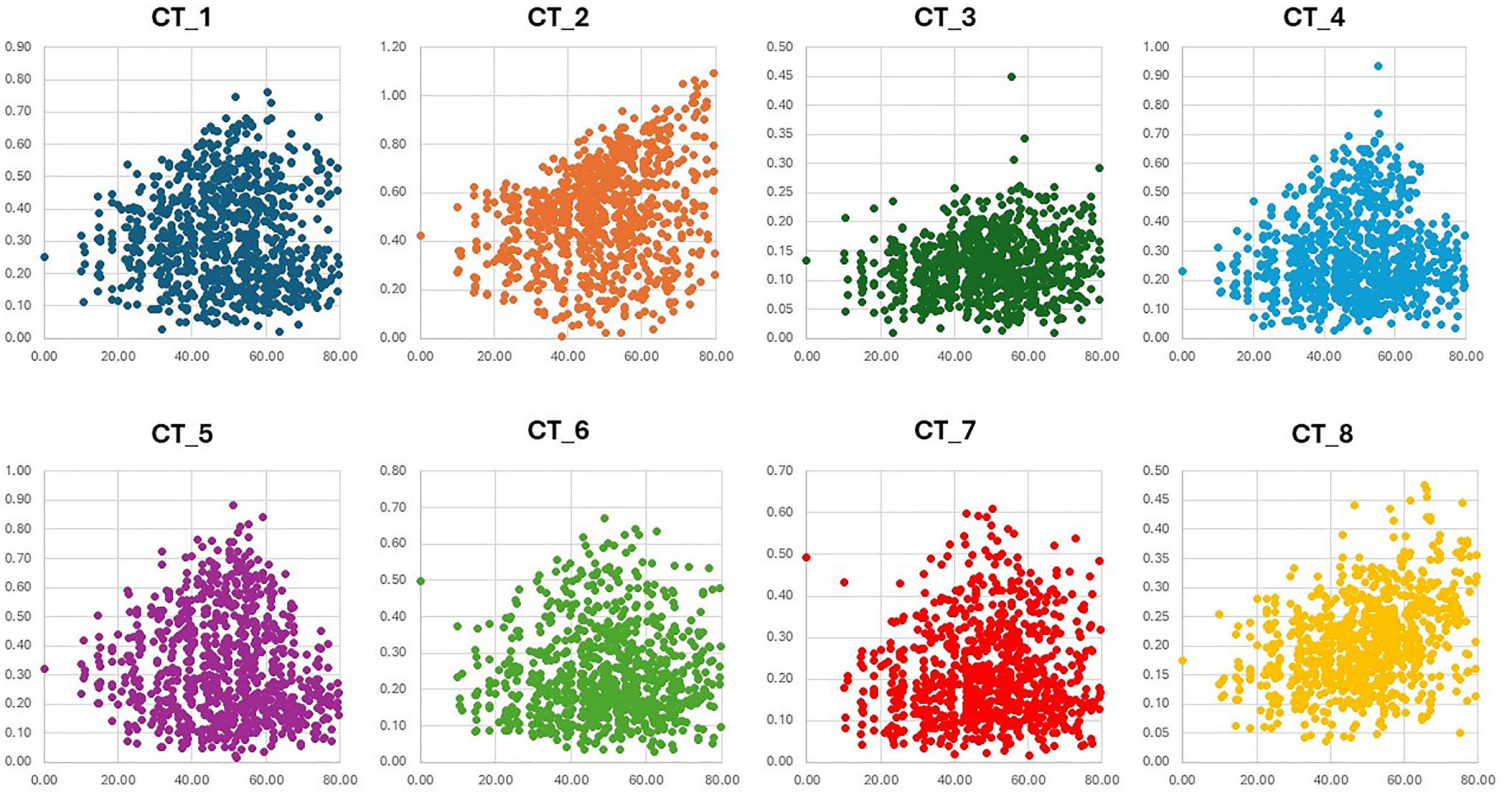
Scatter plots of distortion magnitudes against distance from isocenter for eight CTs, using CBCT as reference. X‐axis: Distance from isocenter (mm); Y‐axis: Distortion magnitude (mm).

In this section, images from eight CT scanners were retrospectively analyzed against both the individual reference scan acquired using CBCT and the CAD model. The mean and max distortion magnitudes of each scanner are listed in Table 3. Additionally, using either the CAD model or CBCT as reference, the geometric distortion magnitudes of the eight included CT scanners were plotted both as scatter plots, as shown in Figure 6 and Figure 9, and contour plots, as illustrated in Figure 7 and Figure 8.

**TABLE 3:** Mean and max distortion magnitudes (ℰ) of the eight CT scanners included in the retrospective analysis.

			Vs. CBCT		Vs. CAD Model	
Equipment ID	Vendor	Use	Mean ℰ (± 0.13 mm)	Max ℰ (± 0.13 mm)	Mean ℰ (± 0.13 mm)	Max ℰ (± 0.13 mm)
CT_1	Siemens	RT Only	0.32	0.76	0.49	1.29 ^*^
CT_2	GE	Radiology & RT	0.52	1.09 ^*^	0.30	0.76
CT_3	Siemens	RT Only	0.13	0.45	0.67	1.78 ^*^
CT_4	Canon	Radiology & RT	0.29	0.93	0.60	1.72 ^*^
CT_5	Canon	Radiology & RT	0.33	0.88	0.82	1.88 ^*^
CT_6	Canon	Radiology & RT	0.24	0.67	0.27	0.58
CT_7	Siemens	RT Only	0.22	0.61	0.25	0.64
CT_8	Siemens	RT Only	0.21	0.48	0.30	0.71

^*^indicates scans with a maximum distortion magnitude > 1.0 mm that would have failed the inhouse QA tolerance for SRT planning.

## 4 DISCUSSION

In this study, by comparing two scans acquired under identical conditions, the uncertainty of the DistortionCHECK™ workflow was determined to be 0.13 mm. Repeated analysis of the same dataset yielded identical results, confirming the inter‐analysis reproducibility of the proposed workflow.

Next, the effects of setup uncertainties (rotational and translational shifts) and scan parameters (FoV and slice thickness) on the detected distortion magnitude were investigated. As shown in Figure 3, small rotational or translational deviations did not result in pronounced differences in the measured distortion magnitude. However, when the phantom was positioned with a 5° rotation, substantial increases in detected distortion were observed. This finding is most likely attributable to limitations of the registration algorithm upon large rotations within the DistortionCHECK™ software. It is also consistent with vendor recommendations, which specify that during scanning, “the phantom is rotated no more than ± 5° with respect to machine axes^21^”. In terms of scan parameters, when other confounding factors were controlled, scans acquired with a larger FoV and thicker slice thickness exhibited slightly greater distortion magnitudes than those acquired with a smaller FoV and thinner slices. This is likely related to reduced registration accuracy and increased sampling error as a result of larger voxel dimensions. Therefore, to maximize registration precision and analytical accuracy, reference scans should be acquired using a small FoV and the smallest slice thickness in clinical settings (e.g., 1.0 mm) to maximize spatial resolution.

Subsequently, the effects of fabrication uncertainty and potential temporal variation of the phantom on the accuracy of distortion analysis were investigated. As shown in Figure 4, measurable differences in marker locations were observed among different phantoms. Consequently, the default CAD template should not be universally applied as the reference scan. Instead, a phantom‐specific baseline may be necessary. Meanwhile, it was observed that changes in the distortion magnitude of the same phantom over a one‐year period are negligible. This finding indicates that for the given CT and scanning parameters tested, geometric uncertainty was stable with time. It highlights the structural stability and robustness of the phantom over time, despite the use of water polymer as the filling material.

Next, the geometric distortion magnitudes of two imaging modalities commonly used for image guidance in RT, namely CT and CBCT, were compared. In modern RT, CBCT has been integrated into linac platforms to provide volumetric datasets for image guidance.^25^ Previous studies have demonstrated that the spatial accuracy of CBCT systems generally remains within 1.0 mm,^24^, ^26^ although a 2019 report suggested that the accuracy of CBCT reconstruction algorithms may degrade with increasing distance from isocenter, resulting in distortion and resolution degradations in the longitudinal direction.^27^ To minimize potential confounding factors, the same distortion phantom was scanned using both imaging modalities under identical setups.

As shown in Figure 5, CBCT devices exhibited smaller variations in geometric distortion magnitude compared with CT scanners. This may reflect greater consistency among CBCT systems, or a higher voxel resolution associated with the smaller FoV used in CBCT protocols (0.51 × 0.51 × 1.00 mm^3^ for CBCT vs. 0.78 × 0.78 × 1.00 mm^3^ for CT). From a theoretical perspective, CBCT may also be less susceptible to certain sources of geometric distortion than conventional CT. During CBCT acquisition, the linac couch remains stationary, whereas in CT scanners, inaccuracies in longitudinal couch motion or non‐linear couch bending may introduce geometric distortion.^16^ Furthermore, linacs do not rotate in the cranio‐caudal direction and therefore are not affected by potential distortions associated with gantry tilt. In contrast, for CT scanners where gantry tilt is allowed, existing CT QA protocols permit a relatively broad gantry tilt tolerance of ± 1°.^17^ Although CBCT systems on linacs share certain sources of geometric distortion with CT scanners, such as detector panel sagging and the finite size of the X‐ray source,^28^ linacs used for SRT treatments typically undergo geometric QA with substantially tighter tolerances than CT scanners,^29^ thereby allowing for greater confidence in the accuracy of their imaging systems. However, although the geometric distortion magnitudes observed across CBCT devices were consistently smaller than those observed in CT scanners, this does not necessarily imply that CBCT provides superior spatial accuracy and can be inherently assumed as the “ground truth,” as the effect of other confounding factors such as setup uncertainty cannot be fully eliminated. Nevertheless, CBCT remains a practical and valuable reference modality for the relative assessment and quantification of geometric distortion in CT imaging.

Subsequently, CT images acquired from different scanners were retrospectively analyzed to quantify geometric distortion. As shown in Table 3, when the phantom‐specific CBCT scan was used as reference, the mean distortion magnitudes ranged from 0.13 ± 0.13 mm to 0.52 ± 0.13 mm, while the maximum distortion magnitudes ranged from 0.45 ± 0.13 mm to 1.09 ± 0.13 mm. Alternatively, if the default CAD model were used as reference, the detected geometric distortion magnitude for a particular CT scanner changes dramatically, with mean distortion magnitudes ranging from 0.25 ± 0.13 mm to 0.82 ± 0.13 mm and maximum distortion magnitudes ranging from 0.58 ± 0.13 mm to 1.88 ± 0.13 mm. The observed discrepancy further underscores the importance of selecting an appropriate reference for geometric distortion assessment.

Importantly, it should be noted that the total distortion magnitude reported for a given CT scanner incorporates multiple sources of uncertainty, including setup errors, fabrication tolerances, and uncertainties associated with the reference dataset, that is, CBCT. Nonetheless, compared with a measurement uncertainty of 0.13 mm, measurable geometric distortion was observed across CT scanners, warranting further consideration. In RT, this source of error should be incorporated into risk assessment and margin design, particularly for treatments requiring submillimeter precision, such as SRT. Moreover, caution is warranted when using CT as the gold standard for quantifying geometric distortion in MRI. For instance, using the CT scan acquired on a CT scanner with a maximum geometric distortion exceeding 1.0 mm may lead to underestimation or overestimation of MRI distortion, depending on the directionality of the MRI distortion. If unrecognized, such discrepancies may adversely affect treatment accuracy in high‐precision RT.

Research investigating the underlying causes of geometric distortion in CT scanners remains scarce. Meanwhile, there are several factors that may contribute to the presence of geometric distortion in CT scans, which can be broadly categorized into mechanical and reconstruction‐related factors. Mechanical factors include: (1) longitudinal couch inaccuracy or couch deflection, where patient weight may induce vertical sagging or lateral tilt as the couch translates through the bore^16^; (2) misalignment between the gantry rotation plane and couch motion axis, sometimes caused by miscalibration of the gantry tilt setting, resulting in shear‐like distortions^30^; and (3) focal spot drift, whereby thermal variations in the X‐ray tube can lead to slight shifts in the focal spot position during acquisition.^31^ Alternatively, Reconstruction‐related factors include: (1) interpolation errors in helical reconstruction, particularly at the periphery of the FoV, which may result in spatial warping or elongation; (2) coordinate system transformations, where the presence of gantry tilt requires affine transformations to map data onto a Cartesian grid, and small inaccuracies in this process may introduce measurable distortion; and (3) the partial volume effect, which depends on matrix size, slice thickness, and FoV.

In practice, the observed geometric distortion in CT images is likely the combined result of multiple interacting factors. To explore potential dominant contributors in this study, a scatter plot of distortion magnitudes was generated for the eight CT scanners using the CAD model as reference, as shown in Figure 6. Across all eight scanners, a clear linear relationship between distance from the isocenter and distortion magnitude was observed, suggesting that longitudinal couch inaccuracy or non‐linear couch sag may constitute the primary contributors to geometric distortion. In contrast, as shown in Figure 9, when CBCT was used as reference, this linear relationship became less pronounced. As reported in the literature, CBCT itself exhibits reduced spatial accuracy in the longitudinal direction with increasing distance from the isocenter.^27^ When used as the reference, these shared directional errors may partially cancel those of CT, thereby attenuating the observed relationship. Therefore, when assessing geometric distortion in any imaging modality, whether it is CT, CBCT, or MRI, careful consideration is required in selecting an appropriate reference dataset, as this choice can significantly influence the interpretation of results.

Another notable finding from the scatter plots is that, regardless of whether CBCT or the CAD model was used as reference, the geometric distortion magnitude of CT at the isocenter (phantom origin) remained consistently non‐zero. This may be related to limitations in the rigid registration process implemented in the DistortionCHECK™ software, although no detailed information regarding this registration process is available.

Collectively, these findings raise concerns regarding whether current QA tolerances are sufficient for RT applications employing submillimeter margins. For example, AAPM TG 66^17^ specifies tolerances of ± 1° for gantry tilt and ± 2 mm for CT tabletop orientation relative to the imaging plane. Both parameters represent potential risk factors for geometric distortion in CT imaging^16^ and can contribute to significant distortion magnitudes throughout the FoV. Furthermore, geometric distortion assessment is often performed by measuring the outer dimensions of a phantom with known geometry (e.g., the Wilke phantom),^18^ which, due to limitations in phantom dimensions and analytical approach, fails to provide a comprehensive geometric distortion map across the FoV nor a submillimeter accuracy. It is therefore important for clinicians and medical physicists to recognize the presence of geometric distortion in CT scanners and rigorously quantify its magnitude, so as to inform risk assessment and margin design in high‐precision treatments such as SRT.

This study has several limitations. First, quantification of geometric distortion using the CIRS distortion phantom is limited to a FoV of 250 mm due to the physical dimensions of the phantom. In clinical practice, larger FoV settings may be relevant. Second, although comparative analysis identified that CBCT generally exhibits smaller geometric distortion than CT, neither CBCT nor the CAD model necessarily represents the “ground truth,” thereby introducing uncertainties in the quantification of geometric distortion on CT scanners. Third, although possible causes of geometric distortion are listed, underlying causes of the observed geometric distortion were not systematically investigated. Future studies may benefit from additional experiments, such as deliberately adjusting gantry tilt and evaluating the resulting changes in distortion magnitude, or assessing geometric distortions at different times of the day to evaluate the effect of focal spot drift, to better elucidate the root causes of geometric distortion in CT.

## 5 CONCLUSION

In this study, using the CIRS distortion phantom, the geometric distortion magnitudes of various CT scanners were systematically quantified. The results demonstrated that all eight evaluated CT scanners demonstrated measurable geometric distortion. However, neither the testing methodologies nor the specified tolerances within the current CT QA protocols are sensitive enough to detect submillimeter distortion. Accordingly, clinicians and medical physicists should be aware of the presence of geometric distortion in CT imaging and undertake careful quantification of its magnitude to inform risk assessment and margin design, particularly for high‐precision RT treatments such as SRT.

## AUTHOR CONTRIBUTIONS

Yunfei Hu and Marius Arnesen conceived and designed the project. Yunfei Hu, Emma Cai, and John Shakeshaft acquired the data. Yunfei Hu analyzed and interpreted the data. Yunfei Hu, Emma Cai, John Shakeshaft, Tina Gorjiara, Mikel Byrne, Tim Markwell, and Ben Archibald‐Heeren wrote and reviewed the article.

## CONFLICT OF INTEREST STATEMENT

Yunfei Hu, Mikel Byrne, and Ben Archibald‐Heeren have received travel costs and honoraria for presenting on behalf of Varian Medical Systems, which is part of Siemens Healthineers. Ben Archibald‐Heeren has received travel costs and honoraria for presenting on behalf of Radformation. Ben Archibald‐Heeren sits on a paid research advisory board with Radformation.

## Data Availability

The data that support the findings of this study are available from the corresponding author upon reasonable request.
